# Function of Deptor and its roles in hematological malignancies

**DOI:** 10.18632/aging.202462

**Published:** 2021-01-07

**Authors:** Mario Morales-Martinez, Alan Lichtenstein, Mario I. Vega

**Affiliations:** 1 Molecular Signal Pathway in Cancer Laboratory, UIMEO, Oncology Hospital, Siglo XXI National Medical Center, IMSS, México City, México; 2 Department of Medicine, Hematology-Oncology Division, Greater Los Angeles VA Healthcare Center, UCLA Medical Center, Jonsson Comprehensive Cancer Center, Los Angeles, CA 90024, USA

**Keywords:** Deptor, Multiple Myeloma, leukemia, Non-Hodgkin Lymphoma, hematological malignances

## Abstract

Deptor is a protein that interacts with mTOR and that belongs to the mTORC1 and mTORC2 complexes. Deptor is capable of inhibiting the kinase activity of mTOR. It is well known that the mTOR pathway is involved in various signaling pathways that are involved with various biological processes such as cell growth, apoptosis, autophagy, and the ER stress response. Therefore, Deptor, being a natural inhibitor of mTOR, has become very important in its study. Because of this, it is important to research its role regarding the development and progression of human malignancies, especially in hematologic malignancies. Due to its variation in expression in cancer, it has been suggested that Deptor can act as an oncogene or tumor suppressor depending on the cellular or tissue context. This review discusses recent advances in its transcriptional and post-transcriptional regulation of Deptor. As well as the advances regarding the activities of Deptor in hematological malignancies, its possible role as a biomarker, and its possible clinical relevance in these malignancies.

## INTRODUCTION

In recent years, the Deptor protein (DEP-domain containing mTOR-interacting protein), also known as DEPDC6 (DEP-domain containing protein 6), with gene ID number Q8TB45, has been described. It is a negative regulator of the mTORC1 and mTORC2 signaling pathways, inhibiting kinase activity of both complexes [1]. The Deptor protein is encoded by the DEPTOR gene located on chromosome 8 in the long arm in the 8q24 region [2], of which at least two isoforms have been reported by alternative splicing [3]. As mentioned, Deptor is a negative regulator of mTORC1 and mTORC2. mTORTC1 is involved in anabolism, growth and proliferation signaling pathways [4]. In addition to the growth factors and nutrients that can activate mTORC1, it can also be activated by other factors such as hypoxia, inflammation, energy deficiency, cholesterol and nucleotides. Therefore, mTORC1, through its activation, promotes metabolism and participates in the synthesis pathways of proteins, lipids and nucleotides, contributing to cell growth and proliferation [4, 5]. Interestingly, the activation of mTORC1 inhibits catabolism, blocking autophagy and lysosomal biogenesis. This allows Deptor, which inhibits mTORC1 activity, to activate catabolism. Similarly, activation of this complex can trigger negative feedback regulation of growth factor signaling pathways by regulating IRS1 (Insulin receptor substrate-1) [6, 7] as well as also of the GRB10 factor (growth factor receptor-bound 10) [8, 9]. This negative regulation by mTORC1-mediated feedback can also be positively regulated by the inhibitory effect of Deptor on mTORC1

mTORC2 integrates the signaling pathways of growth factors that regulate processes such as metabolism, survival, organization of the cytoskeleton and cell mobility [4, 5] Activation of mTORC2 regulates SGK1 (Serum and glucocorticoid (GC) -induced protein kinase-1), PKC-α (protein kinase C-α), and AKT (protein kinase B/AKT) [10]. Much like mTORC1, Deptor plays an inhibitory role in the activation of these pathways dependent on the activation of mTORC2.

Due to its ability to inhibit mTOR pathway, studies in Deptor have been of great interest regarding cancer development and progression. Studies reveal a low expression of Deptor in most tumors [1], with some exceptions such as multiple myeloma (MM), thyroid carcinoma and lung cancer, where its expression is high [11]. So, it is suggested that Deptor has a dual role in human malignancies, acting either as an oncogene or as a tumor suppressor [12].

The focus of this review is to discuss current knowledge of Deptor regarding its structure, function and regulation, as well as its role specifically in hematological malignancies such as Multiple Myeloma, Leukemia and Lymphoma.

## Structure of Deptor

**Figure 1:**
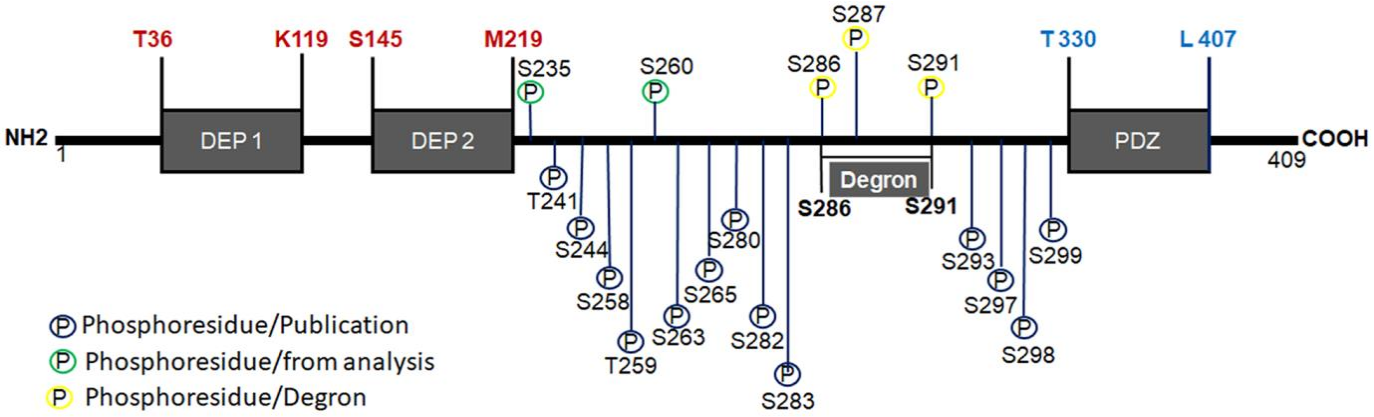
**Structure of Deptor.** Schematic representation of Deptor and his two DEP domains are indicated as well PDZ domain. Degron motif and phosphorylation residues are indicated. phosphorylation at 15 different residues (T and S) located between the DEP2 and PDZ domain junction and comprising between residues T241-S299, which was determined by spectrometric studies, are also indicated.

Deptor is a 409 amino acid protein, weighing 48 kDa and is only expressed in vertebrates [1]. It is a ubiquitously expressed and highly conserved protein that contains two tandems of DEP domains in the amino-terminal region and one PDZ domain at the Carboxyl-terminal region. (Figure 1). The two DEP domains are 84 and 74 aa respectively and are located at residues T36-K119 and S145-M219 respectively of the amino-terminal region [12, 13]. Although their role in these DEP domains is not fully understood, they are believed to play an important role in associating membrane and signaling proteins. These DEP domains have been identified in a large number of proteins [14] and they are highly conserved in sequence and structure. DEP domains function as a membrane anchor [15], signaling regulators at G protein-coupled receptors [14]. Although tandem of two DEP domains are infrequent in proteins containing DEP domains, it’s believed that the tandem of DEP domains may have an important role in mediating the connection to PI3K, just as the inhibitory role of Deptor on mTOR activity can be mediated by the structural organization of these tandem DEP domains [4]. The PDZ domain in Deptor contains 78 aa located in the carboxyl terminal region between residues T330-L407 and is responsible for the protein-protein interaction [1, 13]. This domain is responsible for binding with mTOR, likewise PDZ domains are present in a wide variety of proteins and are highly conserved sequences of G-L-G-F, which participate in folding and interaction with the target protein [16]. Interestingly, some of the proteins that have PDZ domains belong to the Central Nervous System, involved in the neuronal synapse [17]. The PDZ domain can regulate cell signaling by inhibiting phosphorylation of residues in the interaction domain in the carboxyl terminal region on its ligand or in the PDZ domain by itself [16]. Studies by Peterson and Co [1] have demonstrated Deptor phosphorylation at 14 different residues (T and S) located between the DEP2 and PDZ domain junction and comprising between residues T241-S299, determined by spectrometric studies. These studies reveal that the stability of Deptor and its binding to mTOR is in part regulated by Deptor phosphorylation. Additionally, studies by mass spectrometry in our group have identified two additional Deptor phosphorylation residues (S235, and S260), where preliminary studies show that at least one of these residues participates in the stability and degradation of Deptor (Figure 1) [18]. A sequence located in the junction region between the C-terminus of the sequence and the amino-terminal region of PDZ, designated as *Degron* and comprising the S286-S291 region (SSGYFS), has also been characterized. *Degron* is recognized and degraded by βTrCP1, in which the phosphorylation of three sites susceptible to phosphorylation is necessary for their interaction with βTrCP1 (S286, S287 and S291) [19]. This triggers their ubiquitylation and degradation, which may play a role in Deptor function (Figure 1) [20]. Studies have reported that Deptor undergoes ubiquitin ligase-mediated ubiquitination by the SCFβTrCP E3. Upon stimulation of growth factors, Deptor is rapidly degraded by the ubiquitin-proteasome pathway to ensure proper activation of the mTOR pathway [20]. This can be counteracted by the interaction with UBTOR [21], which interacts with the PDZ domain of Deptor, promoting its stability and inhibiting its ubiquitination and consequent Deptor degradation [21].

As we mentioned above, at least two Deptor isoforms originated by alternative splicing have been reported. Isoform 1 has been chosen as the canonical sequence. Isoform 2 differs from the canonical sequence in that it lacks the L42-K142 region (Q8TB45-2) situated in the C-terminal region, encoding a short form of 308 aa (L143-C409) [3]. Therefore, it lacks the DEP1 domain and part of the DEP2 domain, and its possible function is unknown.

As we mentioned above, like another proteins, many of their biological and regulatory functions are controlled by phosphorylation events on Deptor. Since its phosphorylation determines its binding and regulation with the mTOR complex [1], as well as the stability of the protein. To date, at least 18 sites susceptible to phosphorylation of Deptor are known, which suggest of the importance of the phosphorylation events in this protein.

Obviously, the observed phosphorylation events on Deptor as well as other posttranscriptional modification, may have significance in relation to oncogenic pathobiology, such relevance of phosphorylation events has been clearly demonstrated in other hematologic malignances [22–29] It remaining to be seen of these events also regulate contribution of Deptor to carcinogenesis. For example, our recently data, which demonstrate of ERK-dependent phosphorylation of Deptor which maintains its stability, suggests a critical effect in Myeloma. ERK activation by growth factors (i.e., IL-6, EGF-1), as well as mutated RAS, may promote Myeloma progression, in part, via stabilization of Deptor.

## Deptor expression and localization

Important levels of Deptor expression in different tissues have been reported, as well as high levels of Deptor mRNA. An important expression of Deptor in serum, tonsils, bone marrow cell stroma, frontal cortex, spinal cord, stomach, colon, rectum, liver, kidney, spleen, salivary glands, thyroid, adrenal, pancreas, islets of Langerhans, gallbladder, prostate, bladder, skin, placenta, uterus, cervix, ovary, testis, seminal vesicles, as well as in different cell lines, is reported through an analysis of integrated proteomic protein expression (www.proteomicsbd.org) [30]. Of these cell lines, the most important Deptor expression is in breast cancer cell lines (LCC2), Lung cancer (NCI-H522), colon cancer (CCK-81 and HCA-46), cervical cancer (Hela) and multiple myeloma (8226). At the intracellular level, Deptor is expressed in cytosol, mitochondria and nucleus, with less expression in the plasma membrane, cytoskeleton, endoplasmic reticulum, endosome and lysosomes (according to an analysis in COMPONENTS Subcellular location data base: (https://compartments.jensenlab.org) [31] and the Atlas of Human Proteins [www.proteinatlas.org]) [32]. Different studies describe that the location of Deptor correlates with its function [1, 4, 5, 10, 33, 34].

## Deptor regulation

Studies have demonstrated the different regulatory mechanisms of Deptor, including diverse and complicated epigenetic, post-transcriptional and transcriptional mechanisms. Different studies involve the mTORC1 and mTORC2 complex in downregulation of Deptor at the post-transcriptional level (e.g., phosphorylation) [1]. However, recent study has focused on knowing the transcriptional regulation of Deptor.

### Epigenetic factors

Deptor regulation has been associated with epigenetic processes, as reported in rat kidney cells (NRK-52E), in which inhibition of histone methyltransferase EZH2, responsible for the trimethylation of histone H3 lysine 27 (H3K27me3), was related to an increase in Deptor expression [35]. Effect of inhibition of EZH2 on Deptor expression was confirmed in HCT116 colorectal carcinoma cells, in which interfering RNA treatment as well as a specific EZH2 inhibitor resulted in an increase in Deptor expression [36]. On the other hand, it was reported that in HEC1B cells silencing of the arginosuccinate synthase 1 gene, resulted in decreased Deptor expression as a result of altered methylation of histones, suggesting this mechanism as an epigenetic regulation of Deptor [37]. In another study in prostate cancer cells, there was the indication that treatment with the androgen receptor agonist dihydrotestosterone induces a decrease in acetylation of lysine 9 and 14 in histone 3 in the region corresponding to the 4th intron of Deptor and as a consequence, suppresses the expression of Deptor mRNA [38]. Additionally, an interaction between Deptor and Glycine-N-Methyltransferase has been reported [39]. However, the involvement of Glycine-N-methyltransferase as an epigenetic regulator of Deptor needs to be studied.

### Transcription factors

In this review, we focus on reviewing Deptor’s transcriptional regulatory mechanisms.

Notch1: Studies describe a direct regulation of Deptor expression by Notch by mediating binding to NCID in a conserved sequence near the Deptor promoter start codon [40], in which Notch1 induces a high expression of Deptor, resulting in mTORC1 inhibition. This compensates for feedback inhibition of mTORC1 signaling to PI3K and induces Akt phosphorylation at residues S437 and T308, which contributes to AKT activation, a critical factor in T-ALL leukogenesis. The Notch pathway plays an important role in various cellular processes, including stem cell self-renewal, proliferation, and differentiation. Several studies have identified recurrent mutations in hematologic malignancies that make Notch a therapeutic target in acute T-cell lymphoblastic leukemia, chronic lymphocytic leukemia, and mantle cell lymphoma [41].

GR: Other studies suggest that Glucocorticoids, by binding to their nuclear receptor (GR), are capable of inducing Deptor expression [5] where it was shown that elements of response to GC were identified upstream of the Deptor transcriptional initiation site [42], suggesting that Deptor plays a role in GR-mediated inflammation. Interestingly, studies also suggest that nuclear estrogen receptors directly regulate Deptor expression.

ER-α: This has been observed in ER-α positive prostate cell lines where ER-α antagonists represses Deptor expression in these cells [43]. While it has not been established whether ER-α directly regulates Deptor transcription, it is suggested that ER-α may interact with the response elements identified in the Deptor promoter.

VDR: Several response elements for the vitamin D receptor (VDR) have been identified, and since vitamin D inhibits the activity of mTOR [44], it is suggested that VDR can positively regulate the Deptor expression [45].

AR: Studies suggest a reverse effect for nuclear androgen receptors, since AR directly inhibits Deptor expression by binding to androgen response elements identified in an intron of the DEPTOR gene [38].

**Figure 2:**
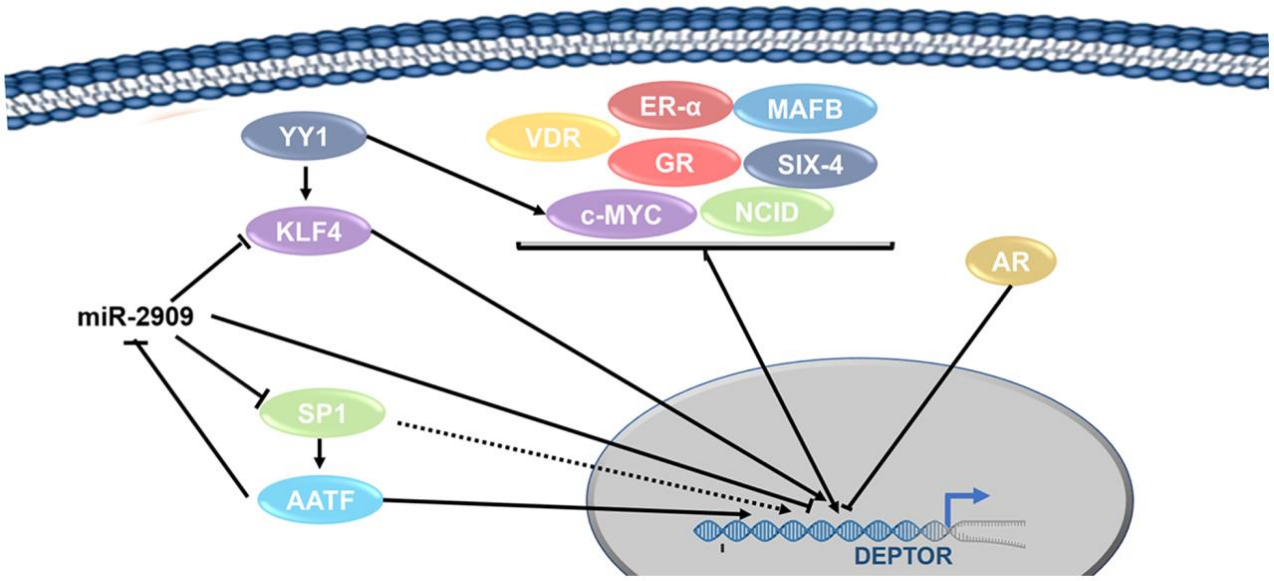
**Transcription factors network involved in the regulation of Deptor.** Transcription regulation of Deptor expression by Transcription factors are represented. Transcription factors involved in the regulation of Deptor revised here are shown.

AATF: Recent studies have described that AATF is capable to regulate the expression of various genes involved in cellular processes. These genes are KLF4, C-Myc, p53, p21, mTORC1 and recently reported Deptor [46–48]. Interestingly, this regulation of Deptor by AATF could be due to the direct binding to its promoter, or it can be down-regulated by regulating the expression of miR-2909, which binds to the 5” UTR region of Deptor by inhibiting its expression (Figure 2) [49].

c-MAF: Studies suggest that the c-MAF proto-oncogene and the MAFB transcription factor who regulates cellular processes are capable of regulating Deptor expression. This happens specifically in MM where high Deptor expression is shown [1], although it is unknown to date whether they do it directly or not.

Six4: Another transcriptional regulator that has been reported to be capable of regulating Deptor is Six4, which is a transcriptional factor involved in cell differentiation, cell migration, and cell survival. Consensus binding sites for Six4 have been identified in the Deptor promoter, and mutations at these sites inhibit activation of Deptor expression by Six4 and its cofactor Baf60c [50].

C-Myc: Additional studies show that Deptor is regulated by Wnt/β-Catenin/c-Myc and has implications for the pathogenesis of colorectal cancer. Where a direct transcriptional regulation of C-Myc on the Deptor promoter is demonstrated (Figure 2) [51].

### Predicted transcription factors in the regulation of deptor

Our group carried out a bioinformatic study using the Jaspar platform (http://jaspar.genereg.net) [52], in order to predict the possible binding of transcription factors in the Deptor promoter. This analysis reveals according to the significant score, that transcription factors such as Arid3a, CDX1, CDX2, CREB1, CUX1, KLF4, KLF14, SP1 BCL-6 and YY1 may be involved in the transcriptional regulation of Deptor (Table 1).

**Table 1:** Predicted transcription factors involved in the regulation of Deptor.

**Transcription Factor**	**Score (Jaspar)**	**Position**		**Predictive sequence (Jaspar)**
		**Start**	**End**	
Arid3a	9.84	-1693	-1688	ATTAAA
CDX1	12.25	-242	-234	ACAATAAAA
CDX2	13.312	-244	-234	GTACAATAAA
CREB1	10.52	-770	-759	CCTGACCTAGG
CUX1	12.19	-1835	-1826	TTATCGATAG
KLF4	11.50	-258	-248	CCACACCCAA
KLF14	14.45	-82	-69	GGCCCCGCCCCCG
SP1	8.59	-81	-72	GGGGGCGGGGC
YY1	8.38	64	69	GCCATC
BCL6	9.29	-1387	-1372	TAACTTTCTAGGCAGA

Predicted transcription factors using Jaspar Platform, with potential binding sites in the Deptor promoter. In this table we list the transcription factors binding sites with the highest score according to Jaspar Score. One or more sites with lower score can be found in the database (Data not shown).

Arid3a: Transcription factor Arid3a is a paralog of the family of interactive AT-rich domains and is associated with the regulation of genes in the development of B cells and cancer such as ovarian cancer [53]. Interestingly we identified at least 6 different possible binding sites for this factor in the Deptor promoter (Table 1). Transcription factor ARID3A emerges as one of the most consistently and differentially expressed genes in ABC-DLBCL, compared to GC-DLBCL [54]. In B-ALL (B-cell Acute Lymphoblastic Leukemia), ARID3a is targeted by miR-125b, where ARID3a inhibits differentiation, increases proliferation and inhibits apoptosis [55]. Recent studies have also identified that miRNA-30b/c/d can regulate key factors of PCD (plasma cell differentiation) such as ARID3A. This miRNA is aberrantly over-expressed in multiple myeloma (MM) tumor plasma cells, suggesting that MM cells frequently acquired expression changes in miRNA that promote dynamic modulation of expression during normal PCD [56].

As mentioned previously, MM shows a high expression of Deptor which is probably regulated by the effect of these miRNAs on the regulation of ARD13A, who could regulate Deptor in MM.

**Figure 3:**
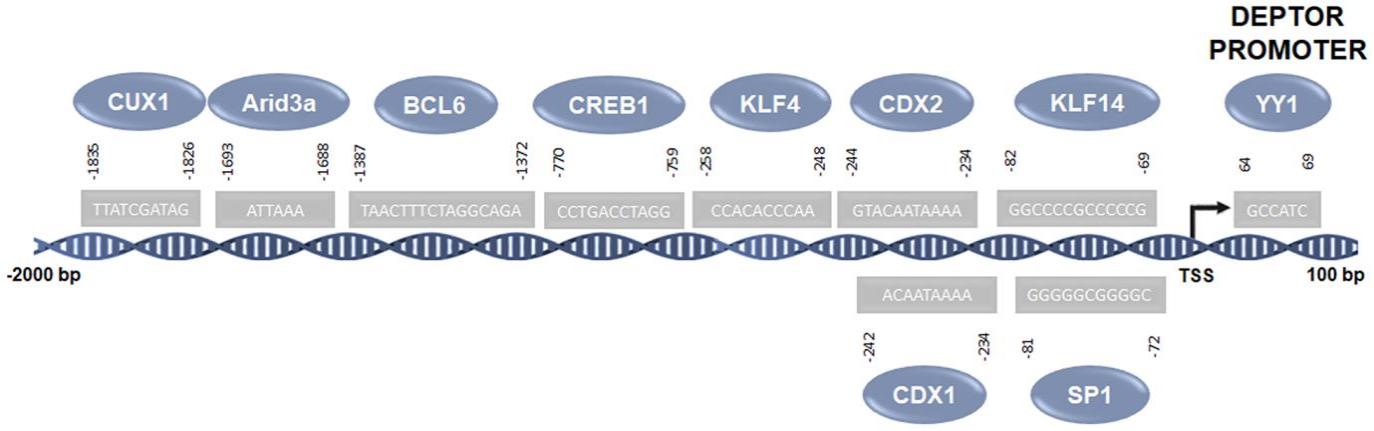
**Predicted transcription factors involved in the regulation of Deptor.** Predicted transcription factors involved in the Deptor regulation according of bioinformatic analysis using the Jaspar platform (http://jaspar.genereg.net) [52].

CDX1: CDX1 is another of the factors identified by our analysis. Interestingly, recent studies indicate that the Wnt/β-Catenin/signaling pathway can regulate CDX1 through CKIP-1 in gastric cancer [57] and as we mentioned previously, Deptor can be regulated by the signaling pathway of Wnt/β-Catenin/ by c-Myc. Therefore, this pathway may also be involved in CDX1-mediated regulation of Deptor from which at least two possible binding sites are predicted in the Deptor promoter (Figure 3 and Table 1). Studies have shown that CDX binding sites are required to mediate Oct-2 ability to activate bcl-2 in lymphomas impacting cell survival [58]. In addition to the role of CDX in hematopoiesis, it has been described that CDX2 can contribute to oncogenesis since data indicates that CDX expression has a functional role in leukemia and malignant blood disease [59].

CREB1: CREB1 is a transcription factor member of the leucine zipper DNA binding protein family which can be activated by the TGF-β signaling pathway [60] which is linked to the regulation of Deptor expression mediated by degradation induced by the mTOR complex. However, a possible transcriptional regulation is suggested to be probably mediated by transcription factors regulated by this pathway. The bioinformatic analysis carried out by our group suggests that CREB1 can regulate Deptor transcription showing two possible binding sites in its promoter (Figure 3 and Table 1). Studies indicate that CREB has a critical role in the proliferation, survival and apoptosis of PBC-ALL cells, suggesting an oncogenic role of CREB in both ALL and CML [61, 62]. Immunohistochemical study shows an important expression of pCREB in DLBCL tissue, suggesting that high levels of pCREB could be associated with tumorigenesis in lymphoma [63]. The presence of CREB has also been associated with resistance to Lenalidomide in MM [64].

KLF4: KLF4 is a transcription factor attributed to bifunctional roles in cancer, as a potent tumor suppressor or as an oncogene [65]. Recent studies have shown that KLF4 can transcriptionally regulate Deptor expression [49]. Interestingly, our bioinformatic study reveals the presence of at least one consensus site for the binding of KLF4 in the Deptor promoter (-82) (Figure 3 and Table 1). KLF4 shows a bifunctional role in hematological malignancies, even though we and other authors have shown an oncogenic role in MM, pediatric lymphoma, and NHL cell lines [66]. Therefore, the regulation of Deptor in NHL and MM could be regulated by KLF4, contributing to pathogenesis at least in MM.

KLF14: KLF14, like KLF4, is a member of the recently identified KLF family of factors and of interest for its involvement in the field of tumorigenesis and immune regulation [67]. KLF14 expression is induced by TGF-β in intrauterine and ectodermal tissue. We identified three consensus binding sites for KLF14 in the Deptor promoter region. Thus, the known TGF-β signaling pathway mediates Deptor regulation, possibly through TGF-β-regulation of KLF14. Preliminary results by our group suggest that KLF14 plays a tumor suppressing role in lymphoma, since a low expression of KLF14 is observed in the more aggressive lymphoma phenotypes.

YY1: YY1 is a zinc finger protein, which regulates numerous genes involved in cell death, cell cycle, cell metabolism, and the inflammatory response. YY1 is highly expressed in many types of cancer which is associated with cell proliferation, survival and metabolic reprogramming [68]. In our bioinformatic analysis we identified at least one YY1 binding site in the Deptor promoter. In addition, studies in lymphoma have demonstrated the transcriptional regulation of KLF4 by YY1 [69]. Therefore, YY1 probably participates in the regulation of Deptor directly on its promoter or through the regulation of KLF4 or c-Myc, which as mentioned above, can also regulate the expression of Deptor. YY1 has been reported to play an important role in several biological processes, including the development and function of B cells [70]. Different studies have shown that YY1 is expressed in acute myeloid leukemia, B-NHL cell lines, and DLBCL tissues [71]. YY1 overexpression has also been reported to correlate with disease progression in MM and childhood acute lymphocytic leukemia [72]. Therefore, YY1 may be playing a role in these hematological malignancies either by direct regulation of Deptor, or by regulating KLF4 and/or c-Myc (Figure 2 and Table 1).

BCL6: The B-cell lymphoma 6 gene (BCL6) encodes a transcription factor belonging to the family Broad-Complex, Tramtrack and Bric-a-brac/Pox and Zinc Finger (BTB/POZ) [73] which is critical for the initiation and maintenance of GC [74, 75]. The Bcl-6 protein is capable of regulating more than 1000 target genes [76]. In normal GCB cells, Bcl-6 also suppresses the expression of the anti-apoptotic oncogene Bcl-2 by binding to its promoter region. However, in DLBCL and FL, Bcl-6-mediated deletion of Bcl-2 is often lost due to Bcl-2 translocation and Miz1 dysregulation [77]. Furthermore, although Bcl-6 is also expressed in Burkitt lymphoma, its role has not been investigated, yet it is expressed in all cases and is likely to contribute to proliferation and survival, since the BCL6 gene has long been recognized as an important oncogene in B-cell lymphoma [78, 79]. Recent studies have identified that certain subpopulations of DLBCL and BL contain MYC, Bcl-2 and/or Bcl-6 translocations and were called “double hit” lymphoma (DHL) or “triple hit lymphoma” (THL) [80]. In the most recent WHO review of lymphoma classification, the DHL/THL category is now recognized as "high-grade B-cell lymphoma (HGBL) with rearrangements of MYC and Bcl-2 and/or Bcl-6. DHL and THL have an aggressive clinical presentation and are difficult to treat with conventional chemotherapy [81, 82]

Interestingly, as discussed below, a high expression of Deptor in BL can be observed, which correlates with a high expression of Bcl-6. Because of this, it would be important to know the possible relation of regulation of Deptor by Bcl-6 in BL.

Since Multiple Myeloma is characterized by clonal accumulation of malignant plasma cells (PC) in the bone marrow, which secrete a monoclonal immunoglobulin, it has been of great interest to know the factors involved in PC differentiation. Recent studies have revealed that alterations in the expression of IRE1, XBP1, FOXP1, PAX5 or BCL6/MTA3 can reprogram PCs to previous stages of maturation [83]. Interestingly, studies in PC have shown that Deptor inhibition changed the transcriptional program associated with PC differentiation through upregulation of PAX5 and Bcl-6, which maintains the B cell program, and downregulation of IRF4, a factor that favors the differentiation of PC [84]. This suggests that Deptor is likely to down-regulate Bcl-6 and according to our analysis *in sílico* (Table 1)*,* where Bcl-6 can regulate Deptor expression, it could be a loop of regulation between Deptor and Bcl-6.

SP1: Finally, our bioinformatic analysis shows at least 4 consensus sites for SP1 binding in the Deptor promoter region. SP1 is a zinc finger-containing transcription factor and its ubiquitous expression apparently mediates the maintenance of normal and malignant biological processes such as cell growth, differentiation, angiogenesis, apoptosis, cell reprogramming, and tumorigenesis [85]. Sp1 exerts its effects on cellular genes that contain putative GC-rich Sp1 binding sites in their promoters. Therefore, it is likely that SP1 can regulate direct Deptor expression. Transcriptional factor SP1 is over-expressed in pediatric acute lymphocytic leukemia B and T, which in turn increases the expression of Bcl-3, MYC, and AATF [48]. SP1 has also been described to be involved in the autocrine-paracrine loop of IL-10 in B-NHL that plays a role in cell proliferation [86]. Recent studies have shown that Sp1 regulates the expression of IQGAP1, which is a protein that plays an important role in ERK-mediated MM cell proliferation [87]. Therefore, it would be interesting to explore the possible Sp1/Deptor interaction network in MM cell proliferation.

This bioinformatic analysis suggests the possible participation of different transcription factors in the regulation of Deptor either directly or through the activation of signaling pathways mediated by growth factors such as TGF-β. However, functional interaction studies should be performed to corroborate the role of these factors in the possible regulation of Deptor (Figure 3).

### Regulation of Deptor by miRNAs

**Figure 4:**
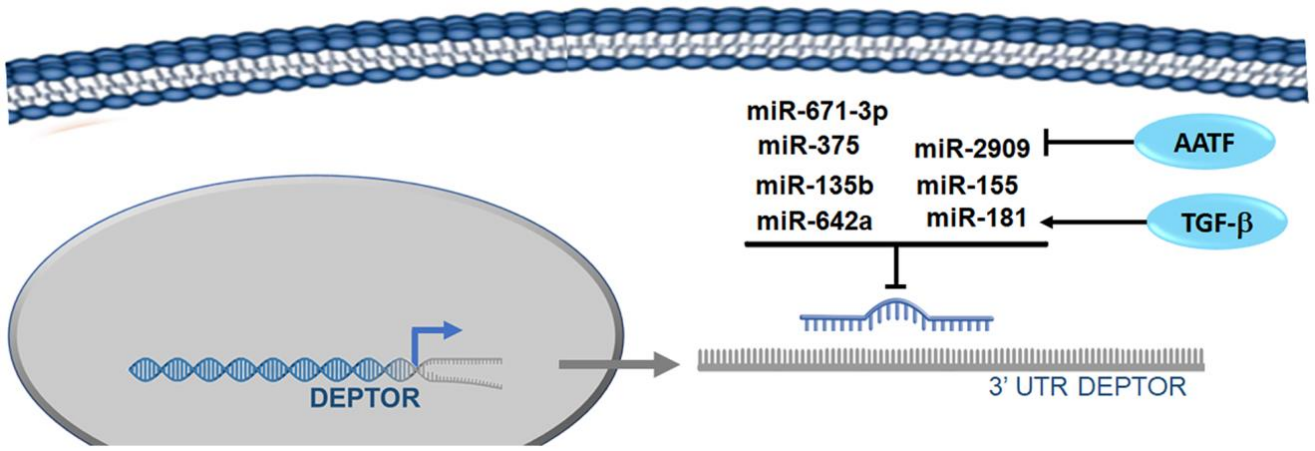
**microRNAs involved in the regulation of Deptor.** Reported microRNAs involved in the regulation of Deptor revised here are shown.

*miR-2909:* Different studies have reported that Deptor regulation expression can be regulated by miRNAs. miR-2909 is capable of regulating crucial genes such as KLF4, c-Myc, p53, p21, SP1 and mTORC1, all essential in the regulation of cellular processes such as cell progression, cell cycle and autophagy/apoptosis [48]. Recent studies demonstrate that the expression of miR-2909 inhibits the expression of Deptor, either by inhibiting the expression of KLF4 that, as mentioned above, positively regulates the expression of Deptor or by direct binding to consensus sites in region 3'UTR of Deptor [49] (Figure 4). As well by the possible regulation of SP1 expression, who can also regulate the expression of Deptor as mentioned above.

As we mentioned, miR-2909 is capable of regulating the expression of KLF4, which in pediatric ALL functions as a tumor suppressor, regulates cell cycle and apoptosis. MiR-2909-mediated down-regulation resulted in loss of KLF4 (isoform 1) activity in B-ALL as opposed to T-ALL where miR-2909 was unable to regulate KLF4 expression in T-ALL (isoform 2), due to mutations in KLF4 3'UTR, which includes the miR-2909 binding site [48]. As reviewed below, Deptor expression is higher in T-ALL than in B-ALL, so these results suggest that downregulation of KLF4 by miR-2909 may be affecting the transcriptional regulation of Deptor by KLF4 in B-ALL as reviewed above [49].

*miR-155*: MiR-155 is a miRNA that has been frequently studied in various types of cancer [88]. The importance of this miRNA in cancer progression is not entirely clear because in some types of cancer miR-155 exerts a tumor suppressing function, such as in the case of melanoma [89]. Recent studies have reported that miR-155 binds to the 3'-UTR region of Deptor by inducing its inhibition in breast cancer, leukemia and bladder cancer cells (Figure 4) [90–92]. Additionally, there is evidence to suggest that miR-155 acts as an "OncomiR", and is important for tumor development, such as leukemia. miR-155 is involved in various physiological processes in the cell, including hematopoiesis, immunity, inflammation and differentiation. Increased expression of miR-155 is observed in many malignant diseases, including lymphomas, acute myeloid leukemia and CLL, where miR-155 has been shown to be involved in the pathogenesis of these lymphoproliferative diseases [93]. Abnormal miR155 expression was closely associated with drug resistance to myeloma, since targeted inhibition of miR155 expression could restore sensitivity to chemotherapy by increasing FOXO3a expression in drug-resistant myeloma cells [94]. Recent studies associate miR-155 expression with increased risk of disease progression in GCB-DLBCL patients treated with R-CHOP [95]. Furthermore, recent studies report that miR-155 promote Burkitt lymphoma progression through PI3K/AKT signaling [96]. However, miR-155 is expressed at low levels in Bortezomib-resistant myeloma cells and is a direct regulator of CD47 through its 3'UTR. In addition, low miR-155 levels are associated with advanced stages of the disease, so miR-155 plays a tumor suppressor role in MM [97, 98]. This bifunctional role of miR-155 in hematological malignancies, as well as in other cancers, does not make clear the importance and relevance in the regulation of Deptor in cancer.

*miR-181*: miR-181 is a family of four miRNAs (a, b, c and d), which is evolutionarily conserved among vertebrates, indicating its important role in physiology. miR-181 was originally described as being highly expressed in T and B lymphocyte cell lines, although its expression is abundant in the lung, brain, and bone marrow [99]. miR-181 acts as a tumor suppressor in acute myeloid leukemia [100]. Recent studies and as previously mentioned have shown that TGF-β negatively regulates Deptor, which increases mTOR activity [101]. However, the mechanism by which Deptor is downregulated by TGF-β was not precisely known. A recent study shows that miR-181a is induced by TGF-β and this miRNA is capable of inhibiting Deptor expression by binding to the 3'UTR region of it. Demonstrating that miR-181a regulates TGFβ-stimulated mTORC1 and mTORC2 activities by direct regulation of Deptor expression (Figure 4) [102]. Recent studies show that miR-181a is overexpressed in MM cells, and may play a role in the biological function of cancer [103]. Additionally, increased levels of miR-181 have been reported in acute myeloid leukemia M1 and M2, and chronic lymphocytic leukemia (CLL) [104, 105]. While other studies in chronic B-cell lymphocytic leukemia (B-CLL) reveal that miR-181 can regulate the T-cell leukemia/lymphoma1 (TCL1) oncogene that is associated to the development of aggressive human B-CLL CD5^+^ [106]. While in cytogenetically normal acute myeloid leukemia (CN-AML), low expression of all miR-181 family members has been identified to have clinically favorable outcomes [107]. Thus, also in AML patients, high levels of mir-181 are expressed in the initial diagnosis compared to the first complete remission [108]. Meanwhile, in Anaplastic large cell lymphoma cells (ALK^+^ ALCL), a low expression of miR-181 is also observed [109].

So although it seems that miR-181 could also have a bifunctional role in hematological malignancies, its role as a tumor suppressor is dominant, which could be associated with its ability to inhibit Deptor expression, where, as we reviewed below, Deptor expression is important in MM and AML, which correlates with low miR-181 expression, while in CLL where significant miR-181 expression is observed, they have low Deptor expression. This suggests that miR-181 is probably playing some role in the regulation of Deptor in these hematological malignancies (Figure 4).

*miR-375*; Recent studies demonstrate that miR-375 promotes osteogenic differentiation in adipocyte-derived mesenchymal stem cells, and one of the mechanisms involved is inhibition of Deptor expression [110]. This study reports at least one consensus site in the Deptor 3'-UTR region for miR-375 binding, thus suggesting that miR-375 is capable of directly inhibiting Deptor expression. miR-375 was identified as a regulator of insulin secretion in pancreatic islets [111]. Subsequent studies revealed that miR-375 participated in multiple biological processes and, furthermore, miR-375 is significantly downregulated in different types of cancer such as multiple myeloma. Additionally, it inhibits their proliferation by inhibiting some important genes such as PDPK1, IGF1R, JAK2, and YAP1 [112–114]. Expression of miR-375 in MM has been related to myelomagenesis and subsequent progression due to its activity on oncogenes such as PDPK1 [115]. Low miR-375 expression levels predict poor outcome in AML patients as it regulates HOXB3 expression [116]. These results suggest that miR-375 in MM and AML may be playing a tumor suppressor role, interestingly in these malignancies there is a high expression of Deptor as reviewed below (Figure 4). Elevated miR-375 expression has been reported in chronic myeloid leukemia [117]. Additionally, in a study in MALT lymphoma, an elevated expression of miR-375 was demonstrated [118]. High miR-375 expression has been associated with the development and progression of pediatric AML, as it correlates with poor relapse-free survival and OS [119]. Recent studies indicate that NFKB2 may be involved in the development of Hodgkin Lymphoma by regulating miR-135a expression [120]. This suggests that miR-375 may be a potent oncomiR in MALT lymphoma, pediatric AML and HL. As reviewed further below, low Deptor expression is observed in CLL, which could be related to high expression of miR-375, while a low expression of miR-375 in MM and AML is related to a significant expression of Deptor in which miR-375 could have a tumor suppressor role through Deptor regulation (Figure 4).

*miR-135b:* A recent study in Multiple Myeloma reveals that Deptor expression is also controlled by two other miRNAs, miR135b and miR642a, which are down-regulated in several MM patients [121]. In other study it was shown *in vitro* that transfection of miR135b and miR642a decreased Deptor levels in myeloma cells. MiR135b and miR642a were observed to modulate Deptor expression through binding to consensus sites in the 3 'UTR region of Deptor [122]. Interestingly, this expression of Deptor regulated by miR135b and miR642a reversed the transcriptional program of myeloma cells and reduced cell size and tumor mass. The miR-135b has been found to play an important role in multiple life activities. For example, miR-135b inhibits the production of LPS-induced reactive oxygen species (ROS), the activation of nuclear factor-κB (NF-κB), and the mRNA expression of TNF-α in human macrophages [123]. In cancer, miR-135b acts as a double-edged factor. It promotes the development of pancreatic cancer and gastric cancer [124]. However, miR-135b can inhibit the proliferation of tumor cells in osteosarcoma [125]. Recent studies show that miR-135b regulates the expression of RBX1, significantly inhibiting the malignant behaviors of MM cells [126]. As we already mentioned, miR-135b is downregulated in MM and is associated with 14q32 rearrangement [127]. In addition, studies reveal the importance of circulating levels of miR-135b in detecting bone disease and in predicting the prognosis of patients with multiple myeloma, suggesting their possible clinical applications [128]. Studies have revealed that short-term treatment of leukemia cells with etoposide, results of transient resistance associated with the transient increase in miR-135b and miR-196b, suggesting that the increase of the expression of these miRNAs, associated with ABC1, may have a role in chemo resistance in leukemia [129]. Studies in Anaplastic Large Cell Lymphoma (ALCL) report that miR-135b is involved in anaplastic nucleophosmin-lymphoma kinase-mediated oncogenicity (NPM-ALK) and potentiates the IL-17-producing immunophenotype [130]. The NPM-ALK oncogene promotes the expression of miR-135b and its target gene LEMD1. At the same time miR-135b decreases chemosensitivity in Jurkat cells, suggesting its contribution to the oncogenic activities of NPM-ALK. In addition, another study describes that miR-135b is over-expressed in ALK^+^ ALCL. Thus, this association of miR135b with ALK expression may represent important downstream effectors of the ALK oncogenic pathway [131]. Interestingly, and as discussed later in the section on the role and expression of Deptor in lymphoma, ALK^+^ALCL vs ALK^-^ALCL, shows in general that there are no differences in Deptor expression. Therefore, it is not clear if miR-135b plays a relevant role in the expression of Deptor in ALK^+^ALCL. Additionally, the expression of miR-135b could play a critical role in the pathogenesis of gastric MALT lymphoma (GML) related to H. pylori infection [132, 133]. Therefore, in addition to the regulation of Deptor by miR-135b, it may also be associated with the regulation of KLF4 who, as previously reviewed, regulates the expression of Deptor [134].

*miR-642a:* There are currently very few reports regarding miR642a and its biological role. Some reports indicated that it is associated with some disease conditions including Leiomyoma, Uterine and Systemic Lupus Erythematosus [135]. As already mentioned, the low expression of miR642a has been reported in patients with MM, especially in the cytogenetic group t (14; 16) [121]. This observation suggests a possible link between the miR-642a downregulation in these MM subsets and an advantage in myeloma cell growth. Also, as previously stated, miR-642a inhibits the expression of Deptor in MM (Figure 4), which leads to the regulation of IRF4, regulating the terminal stages of differentiation [122]. miR642a was downregulated in classical Hodgkin Lymphoma compared to normal B cells, although no difference was found between stage, response to treatment, Disease Free Survival, and Overall Survival. However, there was no clinical association between clinical variables and with miR642a expression. Therefore, more studies are required to evaluate the miRNA profile and the clinical result in Hodgkin Lymphoma [136]. However, as we review below, the low expression of miR-642a in HL may be associated with high levels of Deptor expression in HL (Figure 4).

*miR-671-3p:* A recent study showed that miR-671-3p is capable of regulating Deptor expression in breast cancer (Figure 4) [137]. In breast cancer, it was observed that there is a significantly lower miR-671-3p expression than in normal breast epithelial cells. The forced expression of miR-671-3p induces a decrease in cell proliferation and invasion, whereas inhibition of miR-671-3p obviously promoted cell proliferation and invasion. Deptor was shown by a reporter plasmid to be a target gene for miR-671-3p and overexpression of miR-671-3p caused significant downregulation of Deptor protein expression. These findings demonstrate that miR-671-3p suppresses the proliferation and cell invasion of breast cancer by directly regulating the expression of Deptor [137].

Recent studies have reported that miR-671 plays an important role in multiple cancers [138–141]. The miR-671 precursor forms two mature miRNAs known as miR-671-5p and miR-671-3p. A recent study has shown that miR-671-3p is an antioncogene in breast cancer, where forced expression can inhibit cell proliferation and invasion and sensitize cells to chemotherapy in a Breast Cancer cell line [139]. Additionally, it was demonstrated that the tumor suppressor role of miR-671-3p in breast cancer is given through its influence on the Wnt signaling cascade [140].

However, in non-small-cell lung cancer, miR-671-3p has been shown to play an oncogenic role by inhibiting FOXP2. Therefore miR-671-3p has also been proposed as a potential therapeutic target in NSCLC [141]. Additionally, in glioma, miR-671-3p has been shown to promote cell proliferation and migration *in vitro* and to decrease apoptosis through CKAP4 regulation [142]. This study demonstrates that miR-671-3p is a predominant positive regulator of glioma progression, suggesting that the miR-671-3p/CKAP4 axis may serve as a possible therapeutic target or biomarker in glioma [142].

### Predicted microRNAs in the regulation of Deptor

To address what other possible miRNAs may be regulating the post-transcriptional expression of Deptor, we looked for miRNAs-Deptor interaction in a database (mirTarBase platform; http://miRTarBase.cuhk.edu.cn/) [143], using a value of *p=0.05* for miRNAs whose predicted binding sites in the Deptor 3’UTR region. According to the high prediction score, at least 8 miRNAs were predicted to have a binding site in the Deptor 3’UTR region and these sites were consistent in at least four other databases (miRMap, PITA, RNA22 and RNAhybrid). From these, we revised the following: miR-190a-3p, miR-4796-5p, miR-654-3p, miR-5011-5p, miR-1277-5p. miR-4803, miR-384 and miR-375 (Table 2). These miRNAs were validated by the Next-Generation Sequencing (NGS) method on at least one study, so it is necessary to expand the experimental evidence of these miRNAs.

**Table 2:** Predicted microRNAs involved in the regulation of Deptor.

**MIcroRNA**	**Position in DEPTOR 3'UTR**	**Score (miRTarBase)**	**Pairing of microRNAs and 3’ UTR Deptor**
miR-375	5480 - 5498	125	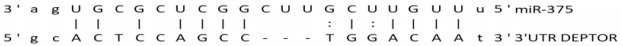
miR-190	1275 - 1299	164	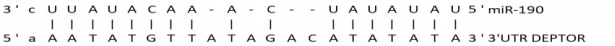
miR-4796-5p	4289 - 4311	152	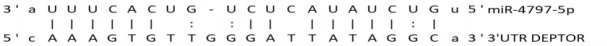
miR-654-3p	4161 - 4183	143	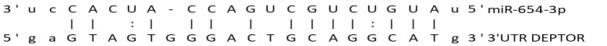
miR-5011-5p	1474 - 1493	155	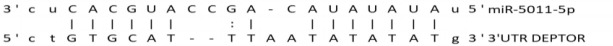
miR-1277-5p	1302 - 1325	194	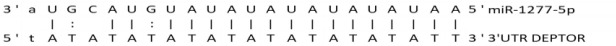
miR-4803	4186 - 4206	135	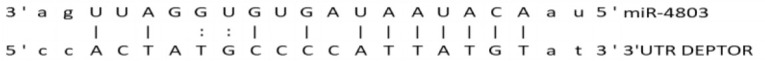
miR-384	2436 - 2455	134	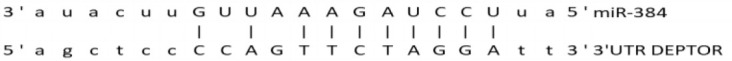

Using the mirTarbase database, we found a potential binding sites of microRNAs in the 3’UTR of Deptor. Here we show the site of miRNA binding, with the highest score in accordance with mirTarbase.

**Figure 5:**
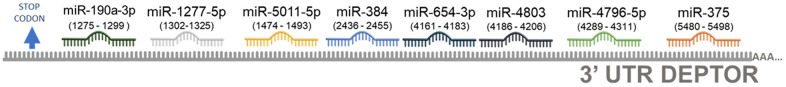
**Predicted miRNAs involved in the regulation of Deptor.** Schematic representation of predicted miRNAS involved in the Deptor regulation using the database (mirTarBase platform; http://miRTarBase.cuhk.edu.cn/) [143]. A value of *p=0.05* for miRNAs whose predicted binding sites in the Deptor 3’UTR region was considerate.

Very little is currently known about miR-190a-3p. Recent studies demonstrate that miR-190a-3p regulates glioblastoma tumorigenesis by regulating PTEN [144]. The possible binding site of miR-190a-3p in Deptor mRNA is shown in Figure 5. The biological role of miR-4796-5p is currently unknown.

Low expression of miR-654-3p has been demonstrated in natural extranodal killer/T-cell lymphoma NKTCL [145]. Recent studies reveal that the expression of miR-654-3p in hepatocellular carcinoma (HCC) is low and is associated with metastasis and is a predictor of poor prognosis. Overexpression of this inhibits the proliferation, migration and invasion of HCC, which proposes miR-654-3p as a tumor suppressor [146]. However, in colon cancer it is observed as an oncomiR, since its high expression correlates with poor survival in these patients [147]. Its role in hematologic malignancies is currently unknown with precision. The role of miR-5011-5p in biological functions of homeostasis or cancer is currently unknown. Studies in Parkinson's disease reveal that miR-1277-5p overexpression correlates with cell viability and inhibition of apoptosis in human neuroblastoma cells [148]. Our bioinformatic analysis predicts that miR-1277-5p has Deptor as a potential target gene (Figure 5). Like other miRNAs identified in our bioinformatic analysis that may have Deptor as target gene, the biological role of miR-4803, in cancer is unknown. Although the functional role of miR-384 in hematologic malignancies is unknown to date, previous studies have shown that miR-384 was abnormally expressed in various tumors. miR-384 can inhibit the proliferation and invasion of tumor cells in tumor tissues such as gastric cancer, colon cancer and liver cancer and in osteosarcoma [149–151]. Furthermore, miR-384 is involved in the phenotypic properties of cancer cells, such as cell proliferation, apoptosis, and the cell cycle, and plays a vital role in the expression of gene products [152]. These studies suggest a potential role for miR-384 as a tumor suppressor. Our bioinformatic analysis suggested that Deptor could be a possible target gene for miR-384 (Figure 5). Interestingly, our bioinformatic analysis shows the miR-375 which, as already mentioned, is involved in the regulation of Deptor [110].

## Role of Deptor in hematological malignances

### Deptor in Multiple Myeloma

Multiple Myeloma (MM) is a hematologic disorder which is characterized by a proliferation of malignant monoclonal plasma cells in the bone marrow (BM) and/or extramodular sites. Progress in the first PFS and overall survival (OS) has been achieved through the introduction of high-dose therapy (HDT) with autologous stem cell transplantation (SCT), and by the introduction of thalidomide, bortezomib and lenalidomide. Despite the recent progress in OS rates, MM remains an incurable disease and the majority of patients will relapse and will require treatment [153]. Recently studies have been shown that Deptor can be implicated in the chemosensitivity to melphalan *in vitro*. Deptor knockdown was associated with melphalan-induced growth inhibition due to an induction of apoptosis associated with the inhibition of p-AKT protein level, probably due to the fact that Deptor acts as an oncogene, compensating for the inhibition of feedback from p-P70S6K to PI3K, thus activating to AKT [154]. It is known that the inhibition of Deptor results in the inhibition of proliferation and induction of apoptosis in myeloma cells. In addition, high levels of Deptor protein are predictive of a poor response to thalidomide in myeloma, which indicates that the levels of Deptor expression is important as a prognosis marker for myeloma patients and a possible target for a new therapeutic strategy as a small chemicals inhibitor alone or in combination with conventional treatments [155].

Deptor is a 48 kDa protein that binds to mTOR and inhibits this kinase in TORC1 and TORC2 complexes specifically in MM [156]. Different reports have associated Deptor with different pathways and mechanisms in myeloma. Interestingly, Deptor expression is required to maintain myeloma cell differentiation and that high level of its expression are associated with better outcome [122]. Two GEO repository were analyzed in a study [121, 157], finding that the PFS was significantly longer in MM patients with high expression levels of Deptor that in those with low Deptor expression levels (*p* *<0.05*) [122].

In another study, the silencing of Deptor with a new drug prevents the Deptor-mTOR binding, promoting the activation of MTORC1 and MTORC2 and induction of cytotoxicity, suggesting that Deptor is a potential therapeutic target and implicating the critical role of Deptor and mTORC1 in MM [156]. Additionally, Deptor plays a crucial role in the proliferation pathway. This was related to p21 because the knockdown of Deptor induces p21 expression independent of p53, and p21 knockdown prevents the cytotoxic effects of Deptor silencing [158]. According to this the silencing of Deptor with a shRNA resulted in an inhibition of proliferation an increase in cleaved caspase 3 and PARP. This increase was related to apoptosis induction, chemo sensitization, suppression of autophagy [159], melphalan chemo sensitization effects, and reduced levels of phosphor-AKT. These factors caused an inhibition of PI3K-AKT pathway [154]. In this context, several drugs have been proposed for a theorical treatment, such as Thalidomide. This immunomodulating agent can increase PTEN expression and suppress AKT signaling, which is fundamental for MM development and maintaining [155].

Some authors have proposed Deptor as a tumor suppressor in MM, the author suggest that this due to its regulation on mTORC2, which participates in regulation of NDRG1 through the activation of SGK1, which confers chemo resistance to Bortezomib [160]. Since the decrease in SGK1 reduces the phosphorylation of NDRG1 and promotes the induction of apoptosis or chemo sensitivity to Bortezomib. This suggests that depending on the activation pathways mediated by mTORC2; PI3K/PDK1 or PI3K/AKT and the regulation of mTORC2 by Deptor will depend on the role of Deptor as an oncogene or tumor suppressor [12]. Additionally, low levels of Deptor has been found in several cancers [1]. In conclusion, the information above presented support a possible role as tumor suppressor.

A study analyzed if Deptor is capable of regulating different signaling pathways, cells transfected with siRNA-Deptor were analyzed by a protein/kinases microarray. The results showed that Deptor effectively regulates the activity of mTORC1, but interestingly shows that Deptor affects the phosphorylation and activation of ERK1/2 as well as STAT1, p38α, Paxillin, PLCγ-1 and FAK, but each to a lesser degree than ERK1/2.

In that same study, it was analyzed whether the regulation of these kinases was associated with each other, results showing that Deptor inhibition increases ERK1/2 levels independently of the mTOR pathway. Therefore, these findings indicate that Deptor selectively regulates the endogenous activity of ERK1/2 and mTORC1 through independent mechanisms. Deptor has been suggested to show independent regulatory effects on MAPK signaling pathways [161], which is consistent with reports suggesting that it contains putative ERK1/2 binding and association sites [20, 162]. Therefore, the phosphorylation and degradation mechanisms of Deptor reported mediated by the "*degron*" domain [20, 162] may be a regulatory mechanism in biological processes where Deptor plays a role.

Interestingly, and according to the characteristics of MM of higher secretion of immunoglobulins, the participation and homeostasis of endoplasmic reticulum is critical for the maintenance of MM. Deptor has been identified as a protein able to bind to DNA and woks by regulating the transcription of several genes involved in the maintenance of the endoplasmic reticulum such as ERLIN2, KEAP1, PSEN2 and DERL3 [33]. Since it was demonstrated by chromatin immunoprecipitation (ChIP) analysis, that Deptor is capable of binding DNA specifically to the promoter region of these genes.

This topic is still unclear because some reports identified no association with activation of the unfolded protein response after MM cytotoxicity was induced by the silencing of Deptor with drugs. However, Deptor depletion with siRNAs can induce endoplasmic reticulum stress and synergizes the effect of the proteasome inhibitor bortezomib in MM cells [33]. This could be explained as the silencing of Deptor with shRNA produces a feedback downregulation of the IGF/IRS-1/PI3-K/PDK1 pathway [163]. In our group, a set of small chemical inhibitors for Deptor was developed in which they inhibited Deptor/mTOR binding. Therefore, mTORC1 is up-regulated, promoting feedback down-regulation of the IGF/IRS-1/PI3-K/PDK1 pathway, and activating AKT phosphorylation. On the other hand, elimination of AKT decreases activation, and this effect probably results in increased mTORC2 activity which overcomes any inhibitory effect on the PI3-K/PDK1 pathway [163]. These compounds activate mTORC1 and selectively induce MM cell apoptosis and cell cycle arrest [163].

Leucine-rich repeat containing 4 (LRRC4), also called netrin-G ligand-2, is a member of the leucine-rich repeat (LRR) superfamily [164]. Previous studies have confirmed that LRRC4 plays a central role in the development and early differentiation of the nervous system, especially during synapse formation [165]. Furthermore, a new role for LRRC4 as a tumor suppressor for glioma has been reported [166].

Recent studies have shown that LRRC4, an autophagy inhibitor, interacts directly with Deptor, which induces a decrease in the level of Deptor protein. This interaction was determined by co-immunoprecipitation assays for which plasmids with different domains of Deptor were constructed, finding an interaction of LRRC4 with the PDZ region (330-407). Additionally, the deletion of the C-terminal binding domain resulted in an abolition of the interaction of Deptor with LRRC4. This resulted in the activation of mTOR, thus decreasing the level of cellular autophagy [167]. Therefore, it has been suggested that LRRC4 could be used as a possible therapeutic strategy in malignancies associated with high expression of Deptor.

In addition, natural compounds have been proposed as a potential therapeutic treatment for MM; for example, cell lines treated with 1,2,3,4,6-Penta-*O*-galloyl-beta-D-glucopyranoside (PGG) show a cell cycle arrest in G1 phase and increased of apoptosis by caspase 3 cleaved expression. Interestingly, the treatment with PGG in cells was closely associated with a decreased effect on Deptor expression in a dose-dependent way in MM. In contrast with other compounds, this antagonizes the effect of bortezomib [168].

It was reported a Deptor was increased in a time dependent way at 0, 6, 12 and 18 h, and then decreased after nutrient depletion in 8226 cells. These levels were consistent with autophagy [169], confirming the participation of Deptor in apoptosis and provides a possible participation in autophagy.

**Figure 6:**
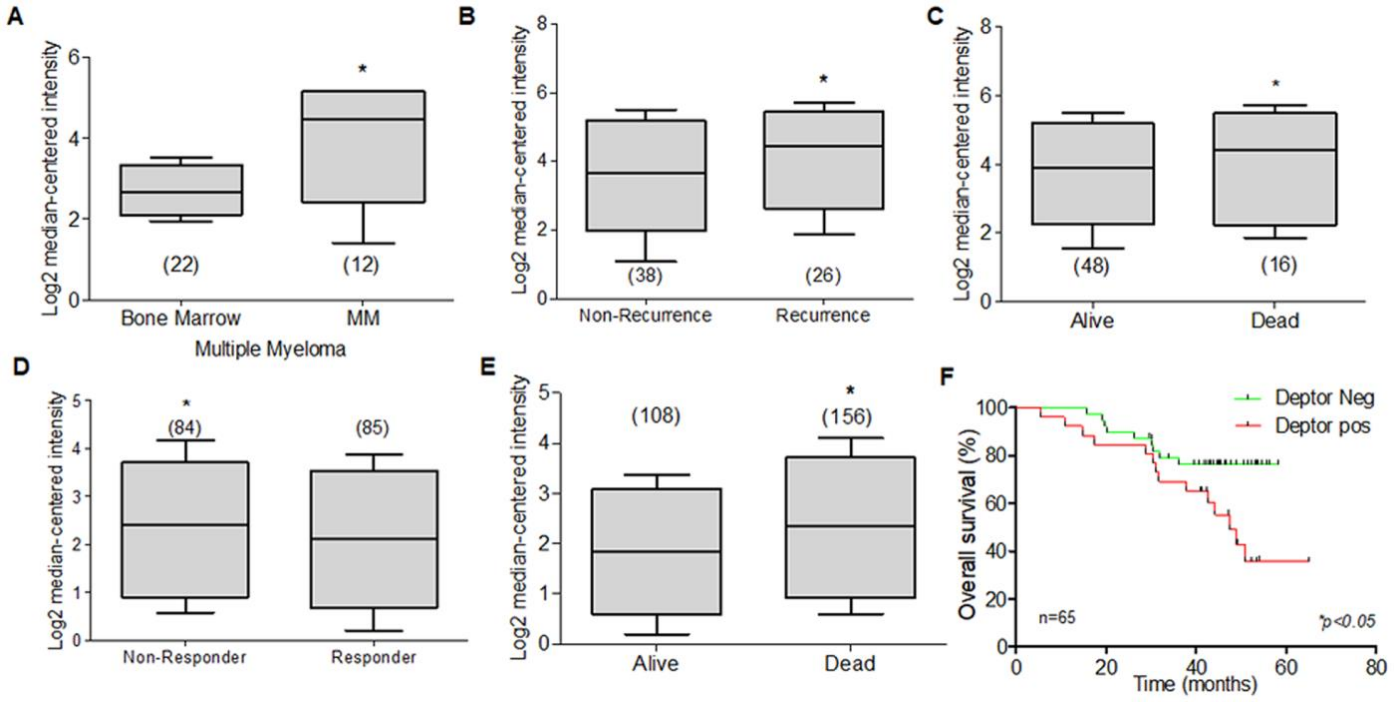
**Expression of Deptor in Multiple Myeloma.** Oncomine Deptor expression was revised in different reports; (**A**) Zhan et al, analysis of Deptor Expression. MM shows a higher expression compared to bone marrow cells (*p<0.001). (**B**) In Carrasco et al, Analysis of Deptor, relationship with disease recurrence and Deptor expression was observed with high Deptor expression compared to non-recurrence (*p<0.05). (**C**) in Carrasco et al Analysis, we found a moderate high expression of Deptor in Alive patients compared to Dead patients (*p<0.05). (**D**) in Mullygan et al, analysis shown a responder patient has a moderated high expression of Deptor compared to Non-responders (*p<0.05). (**E**) In the same data analysis overall survival was shown, and dead patients have an important high expression of Deptor (*p<0.001). (**F**) Overall survival of patients with MM according to Deptor expression. Number of patients (n) is listed next to the graph (*p<0.05).

The potential biological roll of Deptor in MM was predicted by our analysis, using bioinformatic tools (ONCOMINE), and the expression of Deptor was analyzed to be significantly upregulated in MM cells (*P<0.001).* When analyzing different studies related to the expression of Deptor in MM, it is typical to find a high expression of Deptor. In a study involving 133 MM samples, moderate expression was observed compared to plasma cells. In this same study, an analysis was made to observe if there were differences between the expression of Deptor and the stage of the disease and finding that there are no statistically significant differences [170]. Another study shows a significant expression of Deptor in MM (12 samples) compared to bone marrow cells (Figure 6A) [171].

A significant expression of Deptor was observed compared to leukocytes or bone marrow cells in a study that included 84 samples [172]. Studies presented by Chapman that included 289 MM samples [173], Broyl with 320 samples [174], Dickens with 247 samples [172], and finally Zhou with 115 samples [175] showed significant Deptor expression. Interestingly, in two studies that included 65 and 264 samples, respectively, it is observed that there is a moderate relationship with Deptor expression and Progression-free survival (PFS) at 1 year, or with OS (Figure 6B, 6C). In the first study, a relationship with disease recurrence and Deptor expression was observed (Figure 6B). In the second, a relationship with poor response to therapy (Bortezomib) was also observed (Figure 6D) [176, 177]. In a study that included 414 samples, an important Deptor expression is also observed, but it is not possible to establish a significant difference in the relationship with Deptor expression and OS [171]. Another study observed a lower expression in the samples from alive patients of MM compared with a high expression in samples from dead myeloma patients (Figure 6E) [176]. Finally, in the same study using the overall survival, we perform a Kaplan Meier analysis. We compared the overall survival of patients with high and low Deptor expression and observed that high Deptor expression correlates with a lower percent survival (*p<0.05). (Figure 6F) [176].

All these results together strongly suggest that Deptor has a tumor promoting role (oncogene) in MM, where this oncogenic role of Deptor is very specific and important in MM. Additional studies are necessary to establish more clearly the role of Deptor in MM, however, important studies have currently been carried out using Deptor with an important therapeutic target with very successful results that to date have led to pre-clinical studies [18].

### Deptor in Leukemia

Another hematological malignancy in which Deptor plays an important role is Leukemia. Especially in patients with T-ALL, where the role of aberrant NOTCH1 is crucial in the pathogenesis [178]. NOTCH1 promotes leukemogenesis in cooperation with AKT [179]. In this case, NOTCH1 promotes Deptor by union in his promoter identifying a transcriptional control of Deptor and its regulation of AKT, proliferation, and leukemogenesis in T-ALL [40].

In AML, Deptor has been related to KDM4A specifically Depletion of KDM4A decreases [180]. KDM4A is an αKG-dependent enzyme of the Jumonji family of lysine demethylases [181]. KDM4A overexpression reduces the ubiquitination of Deptor while 2HG-induced KDM4A inhibition promotes mTOR activation and act with Deptor. Inhibition of KDM4A results in reduction of Deptor levels, leading to mTOR activation independently of the PI3K/AKT/TSC1-2 pathway [40]. In promyelocytic leukemia cells, the cullin-RING ligase (CRL)-NEDD8 pathway maintains essential cellular processes. The treatment with the antineoplastic ATRA promotes an up-regulated protein expression of Deptor which downregulates mTOR and could promotes the differentiation of promyelocytic cells [182].

**Figure 7:**
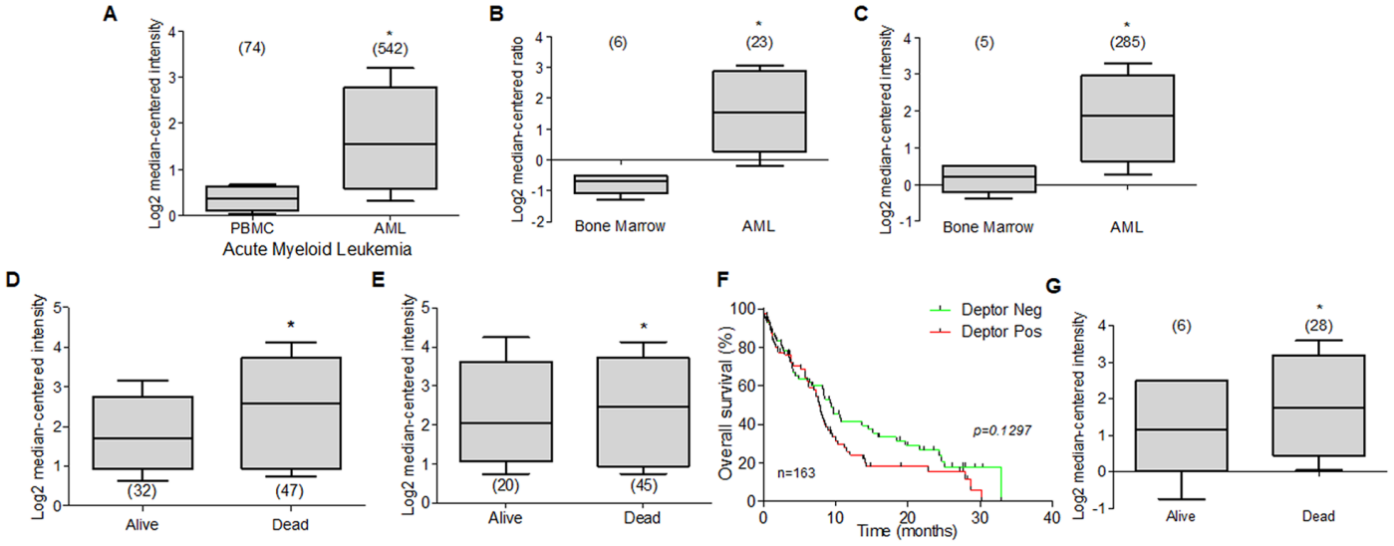
**Deptor expression in Acute Myeloid Leukemia.** Bioinformatic analysis with Oncomine of Deptor expression on AML was revised. (**A**–**C**) three different studies by Hafferlach et al, Andersson et al, and Valk et al, were revised and respectively shows an important higher expression of Deptor in patients with AML compared to controls (PBMC and bone marrow) (*p<0.001). (**D**–**G**) Metzeler et al, and Raponi et al, analysis shown overall survival status on relationship with Deptor expression (**D**, **G**), dead patients have an important high expression of Deptor (*p<0.001). and Progression free survival (**E**), shows a moderate relationship with high expression of Deptor in dead patients (*p>0.05). (**F**) Overall survival of patients with AML according to Deptor expression. Number of patients (n) is listed next to the graph (*^n/s^ p*>0.05).

Currently there is very little information on Deptor expression in leukemia and its biological role. However, we reviewed through Oncomine platform database, studies that included the analysis of Deptor mRNA expression in some types of leukemia. Through this analysis, we found that studies in samples of patients from Acute Myeloid Leukemia (AML) with 542 samples [183], another 23 [184], and 285 samples [185] had a significant expression of Deptor compared to peripheral blood cells or bone marrow cells respectively (Figure 7A–7C). Another study that included 43 samples of AML showed an important expression of Deptor [186]. Another important expression of Deptor is observed in another study that included 162 AML samples, in which analyzing the PFS at 3 years and the OS shows that there is no significant difference between these and the Deptor expression [187]. However, in another study that included 78 AML samples, where important Deptor expression is also shown, it is observed that Deptor expression is related to poor PFS at 3 years and total survival (Figure 7D, 7E) [187].

In the same study, we perform a Kaplan Meier analysis to compare the Deptor expression over percent survival of patients. We observe that high Deptor expression was found in patients with poor survival. (Figure 7F) [187].

Another study reported by Heuser and Co that includes 33 AML samples, a relatively low expression of Deptor is observed. However, when analyzing the response to treatment, a poor response is observed in the group of samples with higher Deptor expression (*p =0.001*), although it is not possible to establish whether there is a relationship between PFS 3-year or OS [188]. In other two studies carried out by Raponi and Co. they show an important Deptor expression in 58 AML samples and other with 34. Interestingly, in the last one, a poor OS is observed in relation to higher Deptor expression (Figure 7G) [189, 190].

In a study in Childhood AML (237 samples), a significant expression of Deptor is shown [191]. However, in another study that included 248 Adult AML samples and 93 Childhood AML samples, a negative expression of Deptor was observed [192]. Therefore, the expression of Deptor in childhood AML is not as clear as the case of AML where an important expression of Deptor is consist in the different studies reviewed here. Acute Myeloid Leukemia (AML) is a hematologic cancer characterized by abnormal clonal proliferation of undifferentiated blasts and by their primary infiltration of hematopoietic organs, such as the bone marrow (BM), lymph nodes, and spleen. Uncontrolled expansion of leukemic cells suppresses normal hematopoiesis [193]. Conventional AML treatment and allogeneic transplantation are the current therapeutic alternatives [193]. However, relapse and disease progression remain the main causes of treatment failure [194]. Thus, treatment of AML remains an important unmet clinical need, especially for patients lacking mutations. According to this review and the important expression of Deptor in AML, this may be of importance in studies as a potential therapeutic target, as in MM (Table 3).

**Table 3:** Expression of Deptor in hematological malignancies.

**Malignancy**	**Subtype**		**Deptor expression**	**Reference**
Multiple Myeloma			High	[170], [171], [172], [173], [174], [175],
Leukemia	AML		High	[183], [184], [185], [186], [187], [189] [190] [191]
	T- ALL		High	[184], [195], [196]
	B-ALL		Low	[184] [183, 195, 196]
	CML		Low	[183]
	CLL		Low	[183]
Non-Hodgkin Lymphoma	DLBCL		High	[208], [209]
			Low	[210–212]
		ABC	High	[208, 217, 218]
		GCB	High	[208, 217, 218]
	FL		Low	[211, 220]
			High	[208]
	BL		High	[209, 211]
	MCL		High	[224]
	MZL		Low	[212]
	ALCL		High	[228]
		ALK^-^	High	[227]
		ALK^+^	High	[227]
Hodgkin Lymphoma	cHL		High	[234]

**Figure 8:**
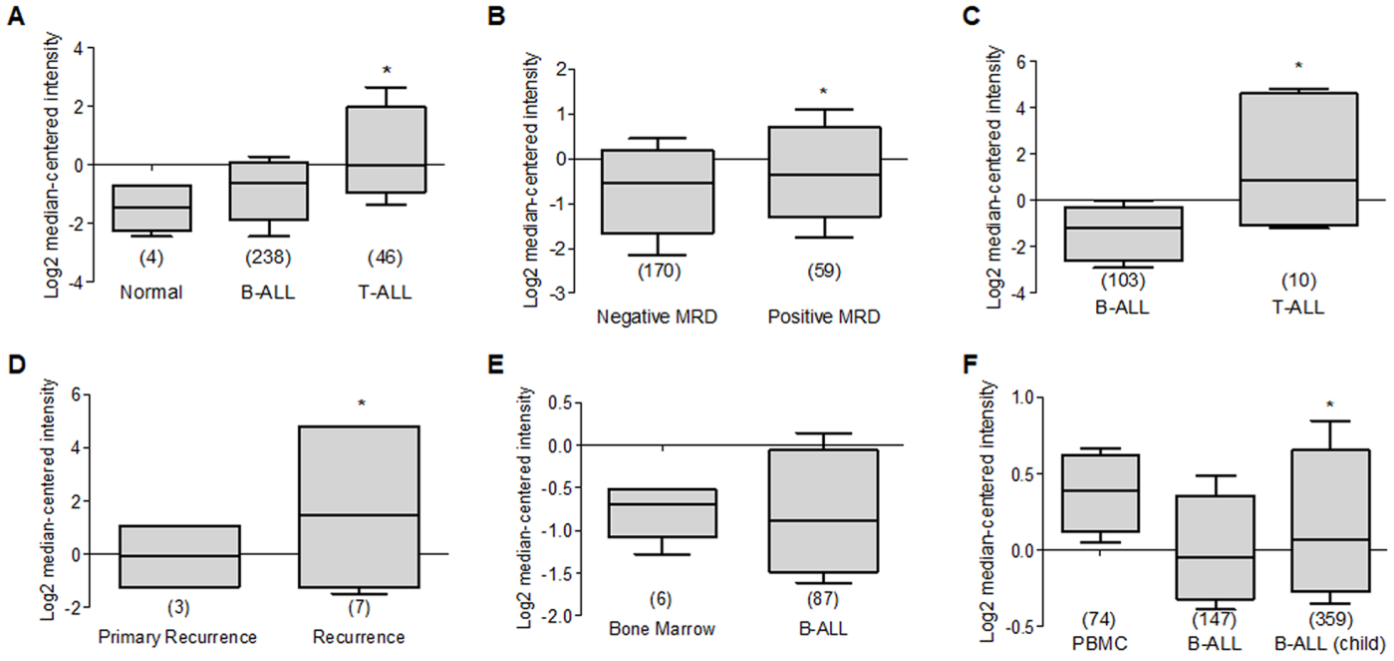
**Deptor expression in Acute Lymphocytic Leukemia.** Oncomine analysis of Deptor expression in ALL was revised. (**A**) in Coustan Smith et al, expression analysis shows a higher expression in T-ALL vs normal cells and B-ALL (*p=0.05). (**B**) analysis of minimal residual disease in relationship with Deptor expression, we observed moderate high Deptor expression in patients with positive MRD (*p>0.05). (**C**) Another study presents by Bhojwani et al, a significant higher Deptor expression was observed in T-ALL compared to B-ALL (*p<0.001), (**D**) In the same study the Deptor expression was related to recurrence of the disease compared with primary recurrence. Moderate high Deptor expression was related with recurrence (*p<0.05). (**E**) On the other study presented by Andersson et al, Deptor expression is low in B-ALL compared with Bone Marrow (*p= N/S). (**F**) On Haferlach et al. study, very low Deptor expression in B-ALL and in B-ALL childhood were observed compared with PBMC (*p= N/S).

When we reviewed the reports that show data in T-Cell Acute Lymphoblastic Leukemia (T-ALL), it is observed in several reports that the expression of Deptor is consistent in this malignancy; for example, in a study with 174 samples, a moderate expression is observed compared to peripheral blood cells [183]. In another report that included 11 T-ALL samples, a high expression of Deptor is observed compared to healthy bone marrow cells [184]. Similarly, in another study in T-ALL with 23 samples, an important expression of Deptor was observed [195]. In other studies where the Deptor expression in T-Cell childhood Acute Lymphoblastic Leukemia was reviewed, it is observed that there is also an important expression of Deptor in a report that included 46 samples, compared to normal cells or B-ALL [196] (Figure 8A). In this study, the minimum residual disease was reported at 46 days and moderate inverse relationship is shown between it and Deptor expression (*p=0.05*) (Figure 8B). Likewise, in another study in 10 samples of T-Cell Childhood Acute Lymphoblastic Leukemia it is observed that the Deptor expression is related to the recurrence of the disease (Figure 8C, 8D) [197]. T-ALL is a highly proliferative hematologic malignancy caused by malignant transformation of T-cell progenitors [198]. T-ALL patients generally present aggressive clinical features correlated with a poor prognosis, including inhibition of normal hematopoietic function [199]. The complete remission rate of T-ALL can reach 94%, and the long-term survival rate can reach 85% [200]. However, 20% of pediatric patients and 40% of adult patients are susceptible to recurrence and develop refractory leukemia [199]. Because of this, understanding the underlying mechanisms of T-ALL development is highly relevant. As already mentioned, a different study indicates that Deptor is importantly expressed in T-ALL and some mechanisms have been described that implicate its role in the development of this condition as an oncogene where it promotes the activity of Notch [40] and activation of the PI3K/AKT pathway. Therefore, all these results suggest Deptor with an important role in the pathophysiology of T-ALL and of interest as an important therapeutic target in T-ALL (Table 3)

Contrary to what was observed in T-ALL, when we analyzed Deptor expression in B-Cell Acute Lymphoblastic Leukemia (B-ALL), a decreased or negative expression is shown. Thus, in a study that included 147 samples of B-ALL, a moderate expression was observed when compared to peripheral blood cells [183]. Consistent with another study that included 87 samples of B-ALL, Deptor expression is very low (Figure 8E) [184]. This low Deptor expression is consistent in Pro-B Acute Lymphoblastic Leukemia (70 samples) [183]. When Deptor expression was reviewed in B-Cell Childhood Acute Lymphoblastic Leukemia in studies that included 359 samples, negative Deptor expression was also observed (Figure 8F) [183]. As already mentioned, ALL is a hematological neoplasm caused by the proliferation and accumulation of lymphoid progenitor cells in the bone marrow or in extramedullary sites, which can be presented as phenotypic subgroups of B cells and T cells. However, more than two thirds of all adult cases are B cell phenotypes [201]. B-ALL has a more favorable survival than T-ALL in a young population and the opposite occurs in older ages [201]. Interestingly, our analysis generally shows significant expression of Deptor in T-ALL and not in those of the B-ALL phenotype, suggesting that Deptor may have a more potential role as an oncogene in T-ALL than in B-ALL. Additional studies are necessary to determine the possible role of Deptor in B-ALL, which according to our review, could play a role as a tumor suppressor much like in other types of cancers, such as breast cancer (reviewed by Caron) [4].

Additional studies also show a negative expression of Deptor in Chronic Myelogenous Leukemia (76 samples), Myelodysplastic Syndrome (206 samples), Chronic Lymphocytic Leukemia (448 samples) [183], while observing a positive expression in Acute Promyelocytic Leukemia (12 samples) [202]. Chronic lymphocytic leukemia (CLL) is one of the most common types of leukemia in western countries [203]. CLL is mainly characterized by the accumulation of mature monoclonal CD5^+^ B cells in lymphoid tissues and peripheral blood [204]. Despite significant advances in its treatment, CLL remains incurable [205]. Therefore, our observations in the analysis of Deptor expression in acute and chronic hematological diseases suggest that Deptor expression is not observed in mature (chronic) phenotype leukemias, but in T-cell lymphoid (acute) leukemias and not so in B cells (Table 3).

Recently, it was revealed that mTOR plays critical roles in regulating CD4^+^T cell activation and proliferation [206]. In addition, Deptor as part of the mTOR signaling complex, may be playing a role in the activation, proliferation and even differentiation of CD4^+^T cells [207]. Deptor has been found to be expressed at high levels in non-activated CD4^+^T cells and its expression regulates the activation state of CD4^+^T cells. Low levels of Deptor are associated with an optimal mTOR activity with a differentiation of CD4^+^T cells [207], while the high expression of Deptor in CD4^+^T cells modulates the differentiation to effector cells and favors Foxp3 T cells. Thus, the mTOR pathway is involved in the differentiation and activation of CD4^+^ T cells and Deptor could be involved in the biological regulation of CD4^+^T cells and their final destination after activation [206]. Therefore, it is not surprising that Deptor is involved in the pathophysiology of hematologic malignancies of T cells.

In conclusion, and according to the studies reviewed here, an important expression of Deptor is observed in T-ALL, as well as in T-Cell Childhood Acute Lymphoblastic Leukemia, while Deptor expression is very low or negative in B-ALL and in B-Cell Childhood Acute Lymphoblastic Leukemia as well as in CLL (Table 3).

### Deptor in Non-Hodgkin Lymphoma

**Figure 9:**
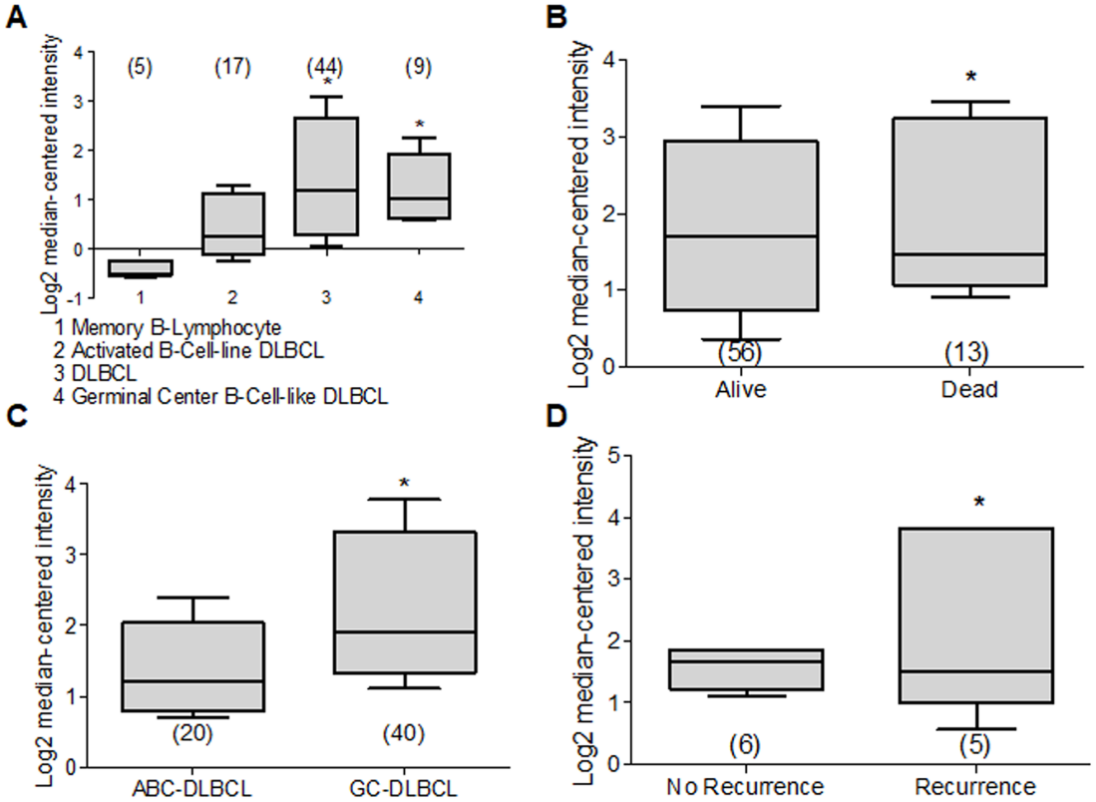
**Deptor expression in Lymphoma.** Based on the Oncomine Analysis we revised Deptor expression in different subtypes of DLBCL lymphoma. (**A**) in Compagno et al. analysis, we observe a differential expression of Deptor in different subtypes of DLBCL. DLBCL, GCB-DLBCL and ABC-DLBCL, shows higher Deptor expression compared with memory B-cell (*p<0.001). (**B**) In Shaknovich et al, Analysis, overall survival according Deptor expression was revised, alive patients show slight high Deptor expression compared with dead patients (*p=N/S). (**C**) in the same analysis two subtypes of DLBCL were revised; ABC-DLBCL vs GC-DLBCL shows a higher Deptor expression in GC-DLBCL (*p<0.05). (**D**) in the same study recurrence was analyzed and Deptor expression was no significantly high in the patients with recurrence vs patients with no recurrence (*p=N/S).

Since there is also very few or no information about Deptor expression in lymphoma and its possible biological role, through Oncomine platform we analyzed different works that contain the mRNA Deptor expression database, finding the following result: A study by Compagno and Co. shows high Deptor expression in Diffuse Large B-Cell Lymphoma (DLBCL) (44 samples) (Figure 9A) [208], which is consistent with another study that included 166 samples [209]. While in other studies, Jais and Co. with 53 samples, Bruce and Co., and Storz and Co. with 6 samples respectively, a negative expression is observed [210–212]. This level of expression of Deptor is important but lower when compared to MM, where it is observed that there is a tendency for greater expression [213].

DLBCL represents the most common Non-Hodgkin’s Lymphoma subtype and is within 30% to 40% of cases in the adult population [214]. DLBCL is an aggressive lymphoma subtype, where two histologically indistinguishable DLBCL subtypes have been identified by gene expression profiling, each derived from a different cell of origin (COO) [215]. The germinal center B-cell-like DLBCL (GCB) subtypes and the other activated B-cell-like subtype (ABC) have been identified. They are controlled by different oncogenic pathways, show different clinical behaviors, and have different clinical results. Therefore, the ABC-DLBCL subtype has worse prognosis and clinical evolution compared to GCB-DLBCL subtype [216, 217]. Because of this, we reviewed studies that contained Deptor expression data in ABC-DLBCL. One study with 17 samples, and others with 20 samples and 167 samples showed a significant expression of Deptor compared with normal memory B cells. In the last study, with 167 samples, an inverse relationship of Deptor expression and poor OS is observed, but is positively related to the presence of extra nodal sites (Figure 9B) [208, 217, 218]. We also reviewed studies that included GCB-DLBCL samples, finding an important expression of Deptor compared to ABC-DLBCL (Figure 9C) [218]. In other studies with 9, 40 and 183 samples respectively, where Deptor expression was analyzed [208, 217, 218], the study with 40 samples shows a moderate inverse relationship between Deptor expression and recurrence at 5 years (Figure 9D) [218].

Follicular lymphoma (FL) is the second most frequent subtype of malignant lymphomas and represents approximately 20% of all cases of lymphoma in the adult population. It is typically an indolent disease with long-term survival [219]. In this review we also analyzed Deptor expression in some studies that contained FL data. We found that in two of the three studies reviewed, one of 191 and the other with 5 samples show a low or negative expression of Deptor [211, 220]. The third study that includes 38 samples shows a high expression of Deptor [208], therefore, considering the size of the samples in general, it can be suggested that the FL shows a low or negative Deptor expression according to this analysis. This is consistent with other study where 8 cutaneous FL samples were included showing to be negative for Deptor expression (Table 3) [212].

**Figure 10:**
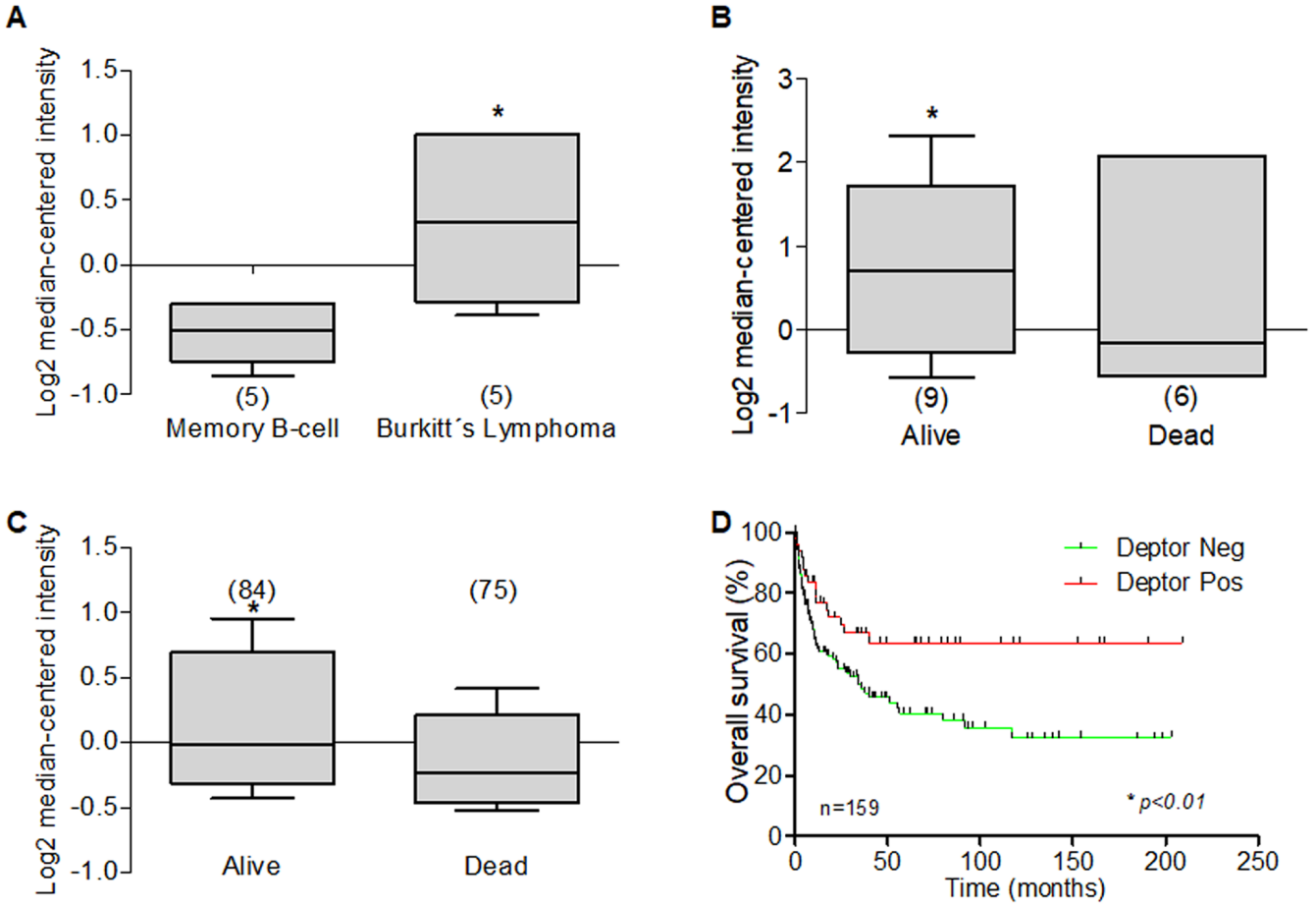
**Deptor expression in Burkitt’s Lymphoma.** Oncomine analysis was done to revised Deptor expression in Burkitt’s Lymphoma. (**A**) in Brune et al, analysis, show high Deptor expression Burkitt’s Lymphoma compared to memory b-cell (*p<0.05). (**B**) In Hummel et al, shows a moderate high Deptor expression the alive patients at 5 years or at total OS (**C**) (*p=N/S or *p<0.05 respectively). (**D**) Overall survival of patients with DLBCL according to Deptor expression. Number of patients (n) is listed next to the graph (^*^p>0.01).

Other frequently diagnosed aggressive B-NHLs include mantle cell lymphoma (MCL) and Burkitt's lymphoma (BL), while another prevalent indolent lymphoma includes marginal zone lymphoma (MZL). In our analysis in this review we found two studies that included 5 and 8 BL samples and are consistent with high Deptor expression [209, 211]. In the study of Brune et al, we observe a high expression of Deptor in BL compared to memory B-cell as control (Figure 10A). Interestingly in the last study, there is an inverse expression between Deptor vs Bcl-2, while there is a positive expression between Deptor and Bcl-6. Furthermore, Deptor expression is inverse related to poor 5-year PFS or OS (*p =0.001*). In addition, a Kaplan Meier analysis was performed to compare survival percent of patients with high Deptor expression. We observed that a high expression of Deptor correlated with a better overall survival percent (*p< 0.01). (Figure 10B–10D) [209]. Bcl-2 is an anti-apoptotic protein member of the Bcl-2 family of proteins that can cause interruption of the apoptotic process and survival of malignant cells [221]. The aberrant expression of the Bcl-2 protein contributes to the pathogenesis of many types of human malignancies, including leukemias and lymphomas. Within B-NHL, Bcl-2 overexpression commonly arises from genetic abnormalities and is substantially different in several lymphoma subtypes. Unlike the rest of B-NHL, the level of Bcl-2 expression in Burkitt lymphoma is low or undetectable, which has been used as part of the diagnostic algorithm for this subtype of lymphoma [222, 223].

Therefore, probably due to the low expression of Bcl2, it was not possible to establish any relationship between the positive expression of Deptor and Bcl-2. Knowing the important role of Bcl-2 in the response to treatment and the inverse expression of Deptor, it is not surprising that the expression Deptor is not related to response to treatment. So, these observations briefly suggest that Deptor may have a role in lymphoma development than in mechanisms of resistance to therapy. BCL6 gene encodes a transcription factor that is critical to the normal development of the B-cell germinal center reaction important for cell proliferation and DNA damage. Bcl-6 activity can be deregulated by a variety of mechanisms and contributes to the development of B-cell lymphoma. Therefore, finding a positive relationship between the presence of Bcl-6 and Deptor may suggest that Deptor has some role in the development of Burkitt’s Lymphoma. Furthermore, our review of the *in silico* analysis of possible transcription factors that regulate Deptor expression is interestingly found in Bcl-6, as described in the Deptor regulation section (Table 1 and Figure 3). Likewise, our analysis shows an important expression of Deptor in 57 MCL samples, which, as already mentioned, together with BL is another of the most aggressive B-NHL subtypes [224]. Therefore, this data suggest that Deptor expression is more related to more aggressive B-NHL subtypes like ABC-DLBCL and GCB-DLBCL. We also analyzed a report of MZL, an indolent B-NHL subtype, and found low and negative Deptor expression compared to tonsil tissue [212]. This seems to be consistent with our hypothesis of low Deptor expression in less aggressive lymphoma subtypes (Table 3).

**Figure 11:**
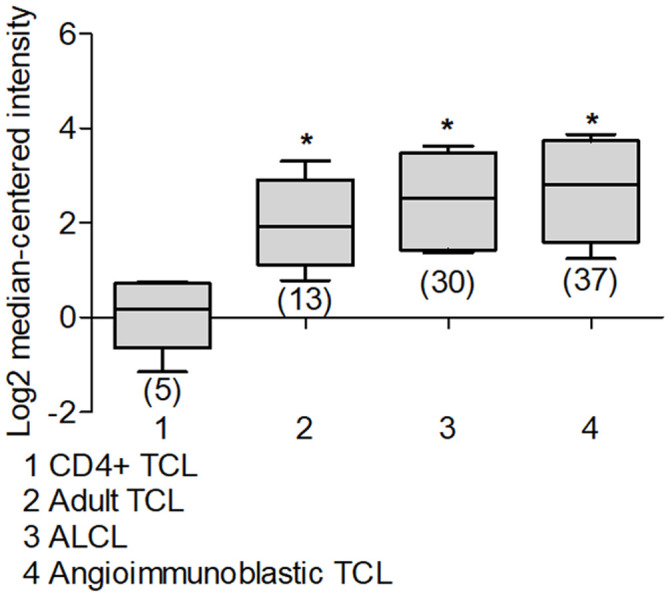
**Deptor expression in T-cell Non-Hodgkin Lymphoma.** Using Oncomine we analyzed Deptor expression on TCL and ALCL. A) a study by Iqbal et al, show high Deptor expression in Adult T-Cell leukemia, Anaplastic Large Cell Lymphoma and Angioimmunoblastic T-cell lymphoma compared with CD4^+^ T cell lymphoma (*p<0.001).

ALK-positive anaplastic large cell lymphoma (ALK^+^ ALCL) is a rare subtype of peripheral T-cell Non-Hodgkin Lymphoma (NHL), and is more common in children and young adults. Patients generally have advanced stage (stage III or IV) disease and systemic symptoms (75%) [225]. Lymphoma cells show strong and uniform CD30 expression and aberrant expression of anaplastic lymphoma kinase (ALK) protein due to translocations involving the ALK gene at the 2p23 locus [80]. ALK^+^ ALCL has a more favorable clinical course than ALK negative cases [226]. Two studies included in our analysis show that there are no differences in Deptor expression between Anaplastic Large Cell Lymphoma, ALK-Negative (4 samples), and Anaplastic Large Cell Lymphoma, ALK-Positive (5 samples) [227], while the other study shows a very high expression of Deptor in ALCL (30 samples) [228]. A study containing 13 samples of Adult T-Cell Leukemia/Lymphoma and 37 samples of Angioimmunoblastic T-Cell Lymphoma was also analyzed, showing that both conditions show a very important expression of Deptor in the samples analyzed (Figure 11) [228]. Adult T-Cell Leukemia/Lymphoma (ATL) is an aggressive type of intractable peripheral T-cell malignancy. It is caused by infections with the human T-cell leukemia virus type I (HTLV-1). The results, after therapeutic interventions for ATL, have not been satisfactory [229], and is characterized by multiple organ invasion by ATL cells, a high frequency of opportunistic infections, resistance to chemotherapeutic drugs, and poor prognosis [230].

Angioimmunoblastic T-cell lymphoma (AITL) is another peripheral T-cell lymphoma with aggressive lymphoproliferative disorders, almost all associated with poor clinical outcomes [231]. The review of our Deptor expression analysis suggests that ATLs generally show significant Deptor expression, although some relationship of Deptor expression with some clinical characteristic cannot be established. Therefore, additional analysis and studies are necessary to establish the importance and role of Deptor in ATLs (Table 3).

**Figure 12:**
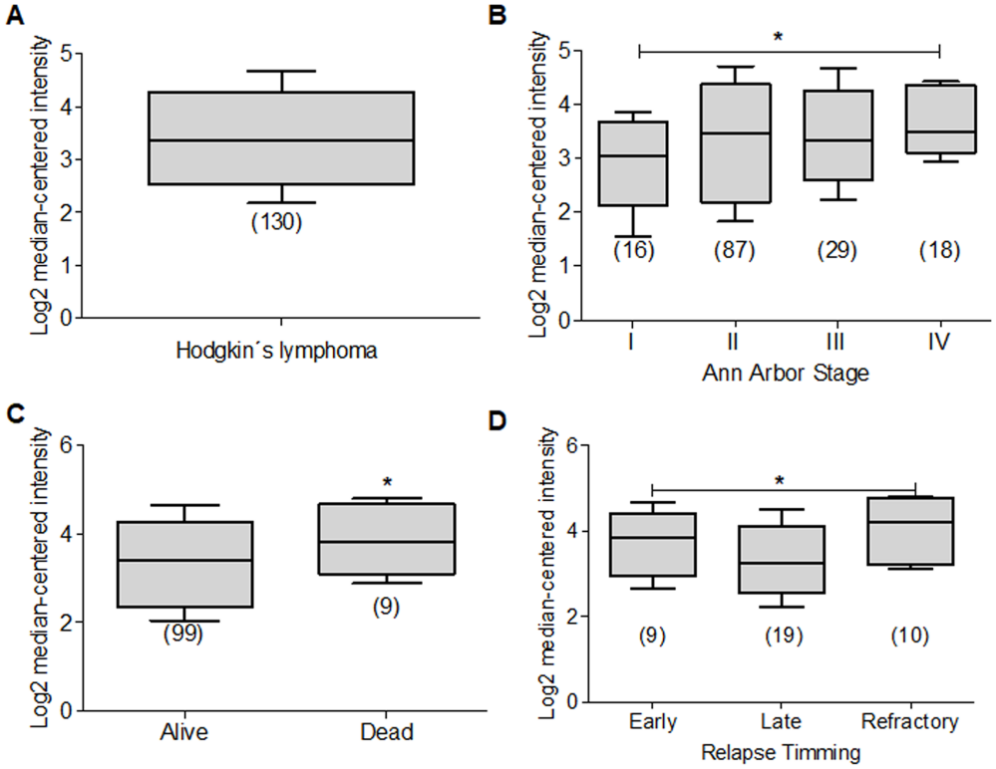
**Deptor expression in Hodgkin lymphoma.** Deptor expression in Hodgkin Lymphoma was revised by Oncomine analyzes. (**A**) Hodgkin Lymphoma shown a high Deptor expression. (**B**) Deptor expression has relationship with stage of the disease, stage IV shown higher Deptor expression compared with stage I (*p<0.05). (**C**) Deptor expression is higher in dead patients vs alive patients when OS was analyzed (*p<0.05). (**D**) Relapse timing was related to Deptor expression in refractory is higher Detour expression compare with late relapse timing (*p<0.05).

Hodgkin Lymphoma (HL) is a lymphoid neoplasm and is one of the most common cancers diagnosed in adolescents. HL is classified according to immunohistochemistry and biological behavior in classical HL (cHL) and predominant HL in nodular lymphocytes. CHL is a more aggressive disease, accounting for 95% of HL cases, while Nodular lymphocyte predominant HL is rarer showing indolent and localized behavior [232, 233]. In a study where Hodgkin's Lymphoma (130 samples) was analyzed, a very important Deptor expression was observed (Figure 12A), and this expression was moderately related to the Ann Arbor stage and to the relapse in the refractory population to treatment (Figure 12B), although there was no relationship with total OS or recurrence. (Figure 12C, 12D) [234]. In another study that included Mixed Cellularity Classical Hodgkin's Lymphoma (17 samples), Nodular Sclerosis Classical Hodgkin's Lymphoma (42 samples), shows an important expression of Deptor, although this is not related to any clinical characteristic such as the outcome [235]. Therefore, according to the review of these analyzes, it is suggested that high Deptor expression in cHL and could be related to the stage and relapse of the disease (Table 3). However, further studies and analysis are necessary to establish the role of Deptor more precisely in cHL.

## CONCLUSIONS

Since the discovery of Deptor in 2009, more than ten years ago, several a large number of information has been accumulated, revealing its importance in a large number of biological processes. As described in this review, Deptor appears to play an important role in the pathogenesis of some types of hematologic malignancies. This is due to its ability to control mTOR activity and PI3K-AKT axis activity, which has been of great importance in cancer among other biological processes. In recent years, Deptor has become an important therapeutic target not only in cancer, but also in metabolic and immunity processes. Therefore, in the coming years the development of many therapies based on the regulation of this protein will not be surprising.
